# A Cross-Cultural Study of Distress during COVID-19 Pandemic: Some Protective and Risk Factors

**DOI:** 10.3390/ijerph18147261

**Published:** 2021-07-07

**Authors:** Ankica Kosic, Tamara Džamonja Ignjatović, Nebojša Petrović

**Affiliations:** 1 Dipartimento di Psicologia dei Processi di Sviluppo e Socializzazione, Facoltà di Medicina e Psicologia, Sapienza—University of Rome, 00185 Rome, Italy; 2 Department of Psychology, Faculty of Philosophy, University of Belgrade, 11000 Beograd, Serbia; tamara.dzamonja@gmail.com (T.D.I.); npetrovi@f.bg.ac.rs (N.P.)

**Keywords:** fear of COVID-19, tendency to worry, social support, distress

## Abstract

Previous studies on the impact of the COVID-19 pandemic on mental health in different countries found an increase in anxiety, stress, and an exacerbation of previous mental health problems. This research investigated some of the protective and risk factors of distress during the COVID-19 pandemic, among which were the perception of receiving social support from family members and friends, and a chronic tendency to worry. The study was conducted in three European countries: Italy, Serbia, and Romania. A total of 1100 participants (Italy *n* = 491; Serbia *n* = 297; Romania *n* = 312) responded to a questionnaire. Results from this study show that distress during the COVID-19 pandemic is higher for people who are chronic worriers and those who have higher levels of fear of COVID-19. More specifically, it is confirmed that a chronic tendency to worry exacerbates the relationship between fear and distress: it is stronger for people who have a greater tendency to worry.

## 1. Introduction

Following the epidemic in Wuhan, coronavirus (COVID-19) soon spread around the world, and thus on 30 January 2020, the World Health Organization declared a Public Health Emergency of International Concern, and on 11 March, officially declared a pandemic. As of March 2021, the COVID-19 pandemic has affected more than 110 million people worldwide, caused more than 2.5 million deaths, and there are approximately 25 million active cases [1]. The disease is mild or asymptomatic in most people; in some (usually the elderly and those with comorbidities), it may progress to pneumonia, acute respiratory distress syndrome (ARDS), and multi-organ dysfunction. The fatality rate is estimated to range from 2% to 3%.

The COVID-19 pandemic and lockdowns have caused widespread concern and fear [2,3,4,5,6]. People are afraid of becoming sick and dying, and are concerned about family members. Several studies in different countries have found a significant relationship between fear of COVID-19 and stress, anxiety, and even depression [2,4,6,7,8,9,10,11]. However, the literature suggests that the potential negative psychological effects of COVID-19 may vary within the population according to some individual and contextual factors [12,13,14]. Some factors may exacerbate the negative effects, whereas others may have a protective role.

Fear of COVID-19 is heightened by information about infection rates, overcrowded hospitals, deaths, and other negative information about the pandemic which are perceived as a risk [15]. The pandemic statistics are available from many types of mass media, but this information changes every day, and it is not always easy to follow and to remember the numbers. In addition, there is evidence that people may suffer from coronavirus news fatigue or apathy, and consequently, they pay less attention to the information about the pandemic [16]. This may explain the fact that, after more than one year of the pandemic, many people still downplay the risks of COVID-19, scoffing at mask-wearing and social distancing. Regardless of these difficulties, people still try to get an idea about the current situation, and they often make estimations about the numbers of positive cases and about how widespread the virus is. These estimates are based on the information they have captured somewhere and on some heuristics. The current study has several aims: (1) to explore whether there is a significant relationship between the knowledge on statistical data and the perception of how widespread COVID-19 is with fear and psychological stress; and (2) to explore if the relationship between the perception of how widespread COVID-19 is in a residential area and fear of COVID-19, on one side, and distress, on the other side, is moderated by some individual factors.

This research takes into consideration several risk and protective factors, and in particular: (1) a chronic tendency to worry; (2) the perception of the possibility of receiving social support from family and friends; (3) the perception of household climate; (4) an individual’s financial situation; and (5) some socio-demographic characteristics (such as age, gender, level of education, and marital status).

We expect that the participants who estimate that there are many positive cases in their residential area will have higher levels of fear and distress. In addition, we expect that the relationship between the perception of how widespread COVID-19 is in the residential area and fear of COVID-19, on one side, and distress, on the other side, may be exacerbated by a chronic tendency to worry [12,17] and negative financial and household situations. Moreover, this relationship may be mitigated by the perception of the possibility of receiving social support [18,19].

## 2. Risk and Protective Factors during COVID-19

The way people respond to stress during the COVID-19 pandemic may depend on several factors, such as socio-demographic characteristics, personality traits, and contextual factors.

Several studies have confirmed the crucial role that social support plays in buffering the negative impact of COVID-19 on mental health [18,19]. Given the fact that, during pandemic, people are obliged to spend most of their time at home, the context in which they live is of crucial importance for their well-being. This study explores the role of perceived social support from family and friends in predicting psychological well-being vs. stress. Normally when people feel distressed, sad, or anxious, they turn to others for social support. Social support is provided by networks that may consist of, for example, family, relatives, friends, neighbors, or coworkers [20]. Several studies have reported that providing and receiving social support is a crucial resource that is associated with a greater resilience to stress [18,19,20,21,22,23], whereas a lack of social support can contribute to distress [24]. We hypothesize that perceived social support from family members and friends may be an important resource in coping with difficulties during this pandemic.

In addition, we expect that a tendency to worry could have a significant impact on distress. Most people worry about the COVID-19 situation and feel that they do not have control over it. Worry has been defined as a chain of thoughts and images, and an anxious apprehension, which are negatively affect-laden and relatively uncontrollable [10,17,25]. Almost everyone worries occasionally, but many people worry every day [26]. The feeling of not being able to control one’s worrying is probably the key to distinguishing between “pathological” and “normal” worrying. Pathological or chronic worry is commonly assessed using the Penn State Worry Questionnaire (PSWQ; [27]), a 16-item inventory that assesses generality, excessiveness, and the uncontrollability aspects of worry [28]. We expect that chronic worry will exacerbate the perception of how widespread COVID-19 is and fear of COVID-19, making people more susceptible to stress. High risk areas of infection or the subjective perception of risk is related to fear, especially in those who worry about other problems, and as such, are more vulnerable.

Furthermore, we expect some socio-demographic factors to have a significant impact, such as gender, age, education level, and marital status. It has been largely demonstrated that women have higher levels of fear of COVID-19, anxiety, stress, and depression than men [3,5,8]. Although the pandemic may affect all age groups, the most vulnerable are children and adolescents, especially those living in an unhealthy family environment [18,29,30] and older people, especially those with health problems [31,32,33].

Furthermore, we expect that the relationship between the perception of how widespread COVID-19 is in the residential area, fear of COVID-19, and stress may be moderated by the perception of an individual’s financial situation and a problematic household climate. Previous studies have found that people with financial difficulties during the COVID-19 pandemic have poorer mental health [30,34]. Many people lost jobs and were weakened financially which created anxiety and stress for them and their families. Moreover, since the pandemic started, a high percentage of people have been asked to work or to attend classes from home, and to limit their social relations with other people to the minimum possible. In relation to this, several studies have reported on negative changes in the mental health of parents and children, and on disruption to the quality of their interactions. For example, an increased frequency of domestic violence and of shouting and physical punishment of children has been registered during the pandemic [35,36,37]. Stress may also be caused by distance from family members, especially when they are severely ill or dying of the virus [38].

Lastly, we also considered trust in governmental institutions as a possible moderator between the perception of how widespread COVID-19 is in a residential area, fear of COVID-19, and stress. Trust in institutions may refer to different aspects [39]. However, some scholars suggest that, despite this complexity, trust judgments may be considered as one-dimensional as different types of judgment combine into one generalized assessment [39,40,41]. Research confirmed that trust in a government’s good intentions and capacity to act well foster a willing compliance with regulations to limit the negative effects of the pandemic [42,43,44,45]. We expect that the individuals who trust the institutions will be less distressed during the COVID-19 pandemic than those who do not trust the institutions.

The current study explores the effects of the above-mentioned factors through a comparative perspective in three European countries: Italy, Romania, and Serbia.

## 3. COVID-19 in Italy, Serbia, and Romania

Italy was the first European country to be severely affected by COVID-19. The virus was first confirmed on 31 January 2020, and it spread quickly throughout the country. On 9 March 2020, the Italian government declared a state of emergency and introduced a lockdown until 11 May 2020 [46]. During that period, Italy registered over 28,884 deaths due to COVID-19, and the number of positive cases was one of the highest in the world [46]. Prior to March 2021, Italy had about three million confirmed cases and almost 100,000 deaths [46].

In the neighboring Balkans, COVID-19 arrived a couple of weeks later, and in Romania, it was confirmed on 26 February 2020. On 21 February, the Romanian government announced a 14-day quarantine for citizens returning from the affected countries (Italy and China in that period). The state of emergency in Romania was issued on 16 March for a period of 30 days [47]. Up until March 2021, the country recorded almost 800,000 confirmed cases and 20,000 deaths in the population [47].

In Serbia, the first positive case was reported on 6 March 2020. The government declared a national state of emergency on 15 March, and adopted containment measures. These included closing borders, prohibiting the movement of citizens during the weekends and between 17:00 and 05:00 on weekdays (and a total ban for senior citizens), suspension of public transport and all activities in parks and public areas, and the closure of commercial activities (except grocery stores and pharmacies). On 6 May, the state of emergency and lockdown were lifted. In response to an increasing number of cases, a state of emergency was declared again on 3 July in several municipalities including the capital, Belgrade. New containment measures were implemented, including restrictions on outdoor and indoor gatherings and the mandatory use of masks in public indoor spaces, which were mostly, but not fully, respected [48]. As of March 2021, almost 450,000 confirmed cases were recorded and more than 4000 deaths in a population of approximately seven million people [49,50,51].

## 4. Materials and Methods

### 4.1. Participants

This study involves the participants from the three European countries: Italy, Serbia, and Romania.

The study conducted in Italy included 491 participants of Italian nationality (*n* = 355 female, 72.7%). From the power analysis we performed (Gpower 3) [52], considering 0.05 as a threshold probability to reject the null hypothesis, and the expected correlations (r = 0.15), this sample size overcame 95% of power, which would require a sample size of 166. The age range was 18–68 years (*M* = 29.44, *SD* = 14.07). The majority of the participants (70.9%) had completed high school, 9.8% had an undergraduate degree, 9.2% had a graduate degree, 3.9% had a post-graduate degree, and 6.3% had completed primary school. The majority (71.1%) of the sample were single, 24.8% were either married or in a relationship, while the remaining were widowed or divorced. Most of the participants (56.4%) were students.

The study conducted in Serbia in the period 20–28 May 2020 involved 297 participants (*n* = 226 female) and the age range was 18–66 years (*M* = 29.29; *SD* = 14.27). The majority of the participants (44.8%) had completed high school, 43.1% had a graduate degree, 10.4% had a post-graduate degree, and 1.7% had completed primary school. The majority (68.7%) of the sample were single, 23.2% were either married or in a relationship, while the remaining were widowed or divorced. Most of the participants (64.0%) were students, and 24.6% were employed.

The study conducted in Romania involved 312 participants (*n*= 255 female) and the age range was18–69 years (*M* = 31.74; *SD* = 10.71). About 20.2% of the participants had completed high school, 44.2% had a graduate degree, and 35.6% had a post-graduate degree. The majority (58%) of the sample were single, 32.7% were either married or ina relationship, while the remaining were widowed or divorced. Most of the participants (62.8%) were employed, and about 25% were students.

### 4.2. Procedure

Data were collected between 20 May and 20 June 2020. This was immediately after the end of lockdown in Italy (18 May 2020). Recruitment was via social media (Facebook) and through students who invited their friends and relatives to participate in the study. The survey was presented as research designed to investigate the psychological impacts of the COVID-19 pandemic. The survey took approximately 15–20 min to complete and it was uploaded on Google Forms (https://forms.gle/oZJzQtMPCaf6gd837 (assessed on 20 June 2020) in Italy, https://forms.gle/PXvWu61DrbyfF17fA (assessed on 20 June 2020) in Serbia, and https://forms.gle/K9S5Ak9xS66995hKA (assessed on 20 June 2020) in Romania).

The response rate was 98% in Italy, and 100% in Serbia and in Romania. The study was approved by the Ethics Committee of the Department of Social and Developmental Psychology, Sapienza–University of Rome (Prot. 468—4 May 2020).

### 4.3. Measures

In the questionnaire, the following groups of measures were used.

Demographics: the participants indicated their age, gender, level of education, marital status (single vs. married/in a relationship), and country and city of residence.

Estimation of the level of spread of COVID-19 in the district: the participants were asked to estimate how many people had coronavirus in their district on a five-point scale (1 = no one; 5 = a large number of people).

Participants also indicated whether they had the coronavirus infection (no–not sure/yes), whether any family member had coronavirus (no–not sure/yes), and whether any friends/acquaintances had coronavirus (no–not sure/yes). At the time of data collection, a relatively small number of participants responded positively on these items, therefore we did not include these variables into the statistical analyses. Moreover, we asked the participants if they knew how many people had contracted coronavirus in their country (approximately), how many people were infected on that day, how many people had died since the beginning of the COVID-19 emergency, and how many people had been infected with coronavirus in their place of residence. We found that a high percentage of the participants did not respond to these questions, or some of them submitted distorted data, thus we did not include any of these items into the statistical analyses.

Economic situation: next, we asked the participants to compare their economic situation with the situation before the COVID-19 lockdown on the five-point scale (1 = much worse; 2 = slightly worse; 3 = more or less the same; 4 = improved slightly; and 5 = much better) and if they were concerned about their economic situation (1 = not at all concerned; 2 = slightly concerned; 3 = somewhat concerned; 4 = moderately concerned; and 5 = extremely concerned). An index of economic difficulties were calculated by summing the responses on the last two items after having reversed the responses on the first item. A higher index indicates greater concern about economic difficulties.

Household climate: the participants rated on a five-point scale (1 = never to 5 = very often) if they were experiencing the following problems in their household during the COVID-19 lockdown: (a) little interaction; (b) sharp discussions and fighting; (c) a lack of respect. An index was created with the higher scores representing a more negative household climate. In addition, we asked the participants if they had been far away from family (partner/children/parents) during the lockdown.

Penn State Worry Questionnaire (PSWQ) [28] the PSWQ is a well-known measure that is free of worry content; that is, it asks about the tendency to worry without identifying the possible targets or contents of those worries. In this research, we used a shortened version consisting of nine items which are rated on a five-point Likert scale (1 = not at all typical of me; 5 = very typical of me). Eight items were worded to indicate pathological worry, with higher numbers indicating more worry (e.g., “Once I start worrying, I cannot stop”), while the remaining item was worded to indicate that worry is not a problem, with higher numbers indicating less worry (e.g., “I never worry about anything”). That item was reversed and a total score was calculated by summing the averaged responses on all items. Higher PSWQ scores reflected greater levels of tendency to worry. The PSWQ demonstrated high reliability in all three countries (Cronbach’s *α* was 0.90 in Italy; 0.91 in Serbia, and 0.93 in Romania).

The social support scale (four items): we asked the participants to rate how confident they were that they would receive emotional support from family members (parents, partner, and children) and from friends and relatives on a Likert scale of five points (1—not at all sure; 5—completely sure). We created two indices of social support: (1) social support from family members; and (2) social support from relatives and friends.

COVID-19 fear scale: we designed a scale composed of five items (I am afraid that I might get coronavirus; I am afraid that I may end up in intensive care because of COVID-19; I am afraid that I might die if I get the coronavirus infection; I am afraid that a loved one might get the coronavirus infection; and I am afraid that someone in my family might end up in hospital because of COVID-19). The participants were asked to rate their level of concern about coronavirus on a five-point scale (1 = not at all; 5 = very much). We ran principle component analysis in order to evaluate the factor structure of the scale and Kaiser’s criterion of 1, and a scree plot was used to select the number of factors. The analysis revealed a mono-factorial structure that explained 69.33% of the variance when considering data of the three samples together (Table S1 in the Supplements). An index was created, with higher scores reflecting higher levels of fear of COVID-19 (Cronbach’s *α* was 0.90 in Italy, and 0.89 in Serbia and in Romania).

Recently, several scales measuring the fear of coronavirus have been proposed in the literature [7,53,54] but were not yet available at the time of this study.

The scale of trust in governmental institutions: we asked the participants to evaluate their level of agreement with three items on a five-point scale (1 = completely disagree; 5 = completely agree). The items were: I believe the government is taking good measures for the prevention and containment of the virus; I trust in the government’s advice on how to prevent the spread of coronavirus; and I think our health system has provided adequate care during the COVID-19 emergency. An index of trust was calculated by summing the averaged responses at these three items. Higher scores reflected higher levels of trust in institutions. The measure had acceptable reliability in all samples (Cronbach’s *α* was 0.73 in Italy, 0.80 in Serbia, and 0.77 in Romania).

The scale of distress: this contained six negative emotional states (sad, frightened, concerned, anxious, distressed, and tense). The participants were asked to rate how they had been feeling lately on a five-point scale (1 = never; 5 = always/usually). The exploratory factor analysis produced a single dimension that explained 61.29% of the variance. An index of distress was calculated and higher scores indicated higher levels of distress (Cronbach’s *α* was 0.87 in Italy and in Serbia, and 0.88 in Romania).

## 5. Results

As a first step, an overall confirmatory factor analysis (CFA) was conducted before proceeding with testing the multigroup measurement invariance. This procedure allows researchers to examine whether respondents from different groups interpret the same measure in a conceptually similar way [55,56,57]. The estimated model consisted of six correlated latent factors (tendency to worry, fear of COVID-19, stress, family climate, family support, and friends support). We adopted the partially disaggregated parcels method, which is achieved by randomly aggregating items that load on the same factor so that there are two or three combined indicators instead of several single-item indicators. The parcels have been shown to have different advantages: they have a higher reliability than single items [58], allow a better fit through the reduction of the variables involved in the model [59], and they may also ensure a more normally multivariate distribution [60]. More specifically, for the tendency to worry, fear of COVID-19, and stress scales, we used two and three indicators, respectively. The model fit indices are satisfactory when running the CFA model across all three countries (χ^2^(67) = 624.416, *p* < 0.001, CFI = 0.915, RMSEA = 0.087).

Moreover, to assess measurement invariance, we ran a multigroup confirmatory factor analysis (MG-CFA) starting from a less restrictive model (i.e., configural) towards more restrictive ones (i.e., metric and scalar) [61,62]. To be specific, configural invariance examines whether items load onto the same latent factor across groups. This model is critically important because one can proceed to testing all subsequent invariance models in the hierarchical sequence only if the configural invariance is achieved. Once configural invariance holds, metric invariance should be tested to warrant that the different groups respond to the items in the same way. Metric invariance means that the factor loading of each item on the latent factor is the same across groups. Satisfying metric invariance demonstrates that the unit and the interval of the latent factor are equal across groups [63]. Thus, it allows the comparison of factor variances and structural relations (e.g., correlations between variables) across groups [64]. Furthermore, when metric invariance is met, scalar invariance is required to assess whether the intercept of each item is the same across groups in addition to the equality of factor loadings [65].

We assessed model fit indices and model comparison fit indices as recommended by [66] and [67]. Results in Table 1 showed a satisfactory fit to the data for both configural (χ^2^(201) = 736.703, *p* < 0.001, CFI = 0.920, RMSEA = 0.085) and metric invariance (χ^2^(209) = 661.429, *p* < 0.001, CFI = 0.932, RMSEA = 0.077). As can be seen, the comparative fit indices (CFI) for both models revealed a good fit, exceeding the suggested cutoff ≤0.90, as well the RMSEA indices, falling into the recommended cutoff ≤0.10. The model comparison test (metric vs. configural) confirmed that metric invariance holds (ΔCFI = 0.012, ΔRMSEA = −0.008). Finally, scalar invariance was tested. The model did not adequately fit the data (χ^2^(225) = 1.215.682, *p <* 0.001, CFI = 0.852, RMSEA = 0.112). The model comparison test (scalar vs. metric) revealed that scalar invariance did not hold (ΔCFI = −0.080, ΔRMSEA = 0.035).

Nevertheless, in summary, based on configural and metric invariance, we demonstrated that the latent structure of all the constructs examined and the factor loading of each item on the latent factor were similar across Italy, Serbia, and Romania.

Descriptive statistics for all variables are shown in Table 2. The assumption of normality of the variables was evaluated and was found to be satisfactory as distributions in all groups were associated with skew and kurtosis less than 2 and 9, respectively. These values are deemed acceptable to demonstrate unimodel distribution [61].

According to ANOVA, most of the variables were significantly different among the three countries (see Table 2 for the Duncan’s post-hoc tests): distress (F(2,1097) = 19.07, *p* < 0.001), fear of COVID-19 (F(2,1097) = 52.07, *p* < 0.001), tendency to worry (F(2,1097) = 24.97, *p* < 0.001), social support from family (F(2,1097) = 22.24, *p* < 0.001), social support from friends (F(2,1097) = 7.77, *p* < 0.001), estimation of widespread COVID-19 in the district (F(2,1097) = 87.86, *p* < 0.001), economic difficulties (F(2,1097) = 16.84, *p* < 0.001), and trust in governmental institutions (F(2,1097) = 31.45, *p* < 0.001). We did not find significant differences for the household climate (F(2,1097) = 1.16, *p* = n.s.). Since our aggregated sample was relatively large, the analysis power was high, and thus it detected all significant differences.

From the means in Table 2, we can see that the level of fear from COVID-19 is significantly, but not drastically, different in the three countries, being the lowest in Serbia and the highest in Romania. It could be associated with the fact that, in that period, the percentage of spread of COVID-19 was lower in Serbia than in Italy and Romania. In effect, we can see that the respondents in Serbia perceived the lowest level of spread of COVID-19 in their district. Instead, the levels of stress and tendency to worry were lower in Romania in comparison to Serbia and Italy. At the same time, the perception of social support from family was higher in Romania than in the other two countries, whereas the perception of social support from friends was higher in Italy than in Romania and Serbia.

The analysis of correlations between the examined variables (Table 3) indicates that, in all three samples, distress was correlated to the highest degree with the tendency to worry, and then with the fear of COVID-19. In addition, we found that distress, tendency to worry, and fear of COVID-19werecorrelated significantly with gender in Italy and Romania, but not in Serbia. In Italy and Romania, male participants had lower levels of distress, fear, and worry than female participants. Furthermore, distress was slightly and negatively correlated with age in all three samples, and fear with level of education only in the Serbian sample. Thus, the level of distress was higher in younger people, and in Serbia, also in those with a lower education.

## 6. Predicting Distress during COVID-19 Pandemic

We conducted a multiple regression analysis using SPSS to examine the percentage of variance in distress accounted for by each of our predictor variables. We considered as predictors some socio-demographic variables (gender, age, level of education, and civic status), index of financial difficulties, the perception of spread of COVID-19 in the place of residence, fear of COVID-19, the perception of social support from family and friends, the perception of household climate, having been distant from family, trust in government institutions, and the tendency to worry (see Table 4). All the variables were standardized before entering the analysis. Furthermore, we considered double interactions between the perception of the spread of COVID-19 in the place of residence with each of these variables: fear of COVID-19, tendency to worry, trust in government, and social support from family and friends. Finally, we included also double interactions between fear of COVID-19 with the variables: tendency to worry, trust in government, and social support from family and friends.

Results in the Italian sample showed that the regression model accounted for a high percentage of variance (51%) (F(22,468) = 22.19, *p* < 0.001). Among the socio-demographic variables, we found a significant effect only of gender (*β* = 0.09, *t* = 2.55, *p* < *0*.01), indicating that females have higher level of distress. Subsequently, we found a significant effect of financial difficulties (*β* = 0.10, *t* = 2.97, *p* < 0.002), meaning that those participants who have economic problems are more distressed. The analysis confirmed that fear ofCOVID-19 is a strong predictor of distress (*β* = 0.20, *t* = 5.18, *p <* 0.001). The tendency to worry is another very strong predictor of distress (*β* = 0.52, *t* = 13.55, *p <* 0.001). We found an effect of interaction between fear of COVID-19 and social support from family (*β* = −0.10, *t* = −2.72, *p* < 0.01), and an effect of interaction between fear of COVID-19 and tendency to worry (*β* = 0.08, *t* = 2.26, *p <* 0.02). We calculated a test to check the multicollinearity, the variance inflation factor (VIF) value and the tolerance statistic. The largest VIF (2.20) was for age, but it was not greater than 10, so it was within tolerance. The corresponding tolerance statistic for age (0.45), was not below 0.1, and again this was within tolerance. Thus, we concluded that multicollinearity did not exist.

From the simple slope analyses, [68] it emerged that the relationship between fear of COVID-19 and distress was stronger for the participants who had a greater tendency to worry (*β* = 0.27, *t* = 5.69, *p <* 0.001) than for those who had lower tendency to worry (*β* = 0.15, *t* = 2.98, *p <* 0.003). In addition, this relationship was stronger when people had less support from family (*β* = 0.56, *t* = 10.29, *p <* 0.001) than when they had more support (*β* = 0.36, *t* = 6.27, *p <* 0.001).

In the Serbian sample, we considered the same variables as in the previous analysis. The regression model accounted for 52.5% of variance of distress (F(22,274) = 13.75, *p <* 0.001) with significant positive effects of financial difficulties (*β* = 0.15, *t* = 3.27, *p* < 0.001), family climate (*β* = 0.11, *t* = 2.25, *p* < 0.03), fear of COVID-19 (*β* = 0.16, *t* = 3.42, *p* < 0.001), tendency to worry (*β* = 0.48, *t* = 10.08, *p* < 0.001), and a negative effect of social support from family (*β* = −0.10, *t* = −1.90, *p* < 0.05). More interestingly, we found an effect of interaction between fear of COVID-19 and tendency to worry (*β* = 0.12, *t* = 2.83, *p <* 0.005). Here also, we checked the VIF value and the tolerance statistic for multicollinearity. The largest VIF (2.42) was for age, but it was not greater than 10, so it was within tolerance. The corresponding tolerance statistic for age (0.41), was not below 0.1, and again, this was within tolerance. Thus, we concluded that multicollinearity did not exist in this analysis either.

In order to better understand the interaction, we conducted a simple slope analysis. We found that the relationship between fear of COVID-19 and stress was stronger and significant only when people had a high tendency to worry (*β* = 0.35, *t* = 5.46, *p <* 0.005), whereas it was not significant when people had low tendency to worry (*β* = 0.01, *t* = 0.17, *p* = n.s.).

Finally, the results on Romanian sample showed that the regression model accounted for 67% of variance of distress (F(22,274) = 25.48, *p <* 0.001). The analysis confirmed once again the significant effect of fear ofCOVID-19 on distress (*β* = 0.35, *t* = 7.88, *p <* 0.001) and of tendency to worry (*β* = 0.59, *t* = 14.11, *p <* 0.001). We also found a negative effect of social support from family (*β* = −0.12, *t* = −2.81, *p <* 0.005). Lastly, we confirmed a significant effect of interaction between fear of COVID-19 and tendency to worry (*β* = 0.08, *t* = 2.19, *p <* 0.03). From the test of multicollinearity, we found the largest VIF (1.78) was for social support from family, but it was within tolerance. The corresponding tolerance statistic for this variable (0.56) was not below 0.1, and again this was within tolerance.

From the simple slope analyses, it emerged that the relationship between fear of COVID-19 and distress was stronger for the participants who had a higher tendency to worry (*β* = 0.37, *t* = 6.30, *p <* 0.001) than for those who had a lower tendency to worry (*β* = 0.24, *t* = 5.13, *p <* 0.001).

## 7. Discussion

This study aimed to explore the role of some protective and risk factors in predicting distress during the COVID-19 pandemic. We considered the perception of how widespread COVID-19 was in the place of residence, fear ofCOVID-19, the chronic tendency to worry, the perception of the opportunity to receive social support from family members and friends, the perception of family climate, the perception of economic problems, and some socio-demographic characteristics (age, gender, level of education, and marital status). This research was conducted in Italy, Serbia, and Romania in the period immediately after the lockdown.

We explored knowledge of the statistics about COVID-19 cases and if that knowledge was associated with fear and distress. We asked the participants to indicate how many people had coronavirus in their country (approximately), how many people were positive at that time, how many people had died since the beginning of the COVID-19 emergency, and how many people had been infected with coronavirus in their place of residence. We noticed that many participants did not respond to these questions, or that some of them submitted distorted data. Very few participants submitted correct answers or something that was close to the correct statistics. This could depend, as mentioned in the introduction, on news fatigue or apathy [19], or simply on the fact that people do not remember data that changes continuously. However, people had some idea about how widespread the virus was, especially in their area of residence, which could more or less correspond to the real situation. From Table 2, we can see that the respondents in Serbia perceived the lowest level of spread of COVID-19 in their district, and consequently, they also had a lower level of fear of COVID-19 than the respondents in Italy and Romania. The official mass media in Serbia had reported on the relatively small number of positive cases at that time, which probably contributed to a lower degree of fear of the pandemic than in the other two countries. In addition, our results confirmed a significant relationship between these estimations and fear of COVID-19 in all three countries.

As expected, people in Italy had the highest level of distress at the time of the study, given the dramatic consequences of the pandemic during the lockdown when Italy had the highest percentage of positive cases and the highest mortality rate in Europe and worldwide. Instead, the level of distress was lower in Romania in comparison to Serbia and Italy, and also the tendency to worry about what could help keep distress under control.

When we look at the correlations (Table 3), we can see that fear of COVID-19 was strongly associated with distress in all the countries considered. The same was also true for the tendency to worry, which is also strongly correlated with fear of COVID-19. We found in all three countries significant correlations between economic difficulties and distress (and with the tendency to worry), and between negative household climate and distress (and again with the tendency to worry).

Consistent with our hypothesis, distress in the countries considered was significantly predicted by the level of fear ofCOVID-19, and above all, by the tendency to worry. It is congruent with suggestions in previous studies that a chronic tendency to worry is a risk factor which strongly contributes to non-adaptive psychological responses to traumatic events and stressors [25,69], as also confirmed in simple slope analyses. In Serbia, the relationship between fear of COVID-19 and distress was significant only for people with a high tendency to worry, whereas in the other two countries, it was also significant when people had a low tendency to worry. It means that the relationship between fear and stress was strong and also existed independently from the tendency to worry.

Social support from family played a positive role in stress reduction in Serbia and in Romania, although not a very strong one, which is similar to the results obtained in several other studies [70]. That effect was not significant in Italy, and it was probably a consequence of the perceived vulnerability within families caused by the first and most dramatic lockdown in Italy. However, we found that social support from family moderated the relationship between fear of COVID-19 and distress. That relationship was stronger when people did not perceive support from family. Furthermore, we found a significant effect of household climate on distress in two samples (Italy and Serbia), but not in Romania. Participants who indicated that household interactions were characterized by conflict and manifestations of contempt felt more distressed.

It has been shown that the level of distress, at least in the case of our study, can be explained very little by the socio-demographic variables considered (age, gender, level of education, and marital status).

## 8. Conclusions

Our primary aim in this research was to explore some of psychological and socio-economic predictors of distress during the COVID-19 pandemic. In addition, we found that fear ofCOVID-19 had a strong effect on distress. What is clear from our three studies is that a tendency to worry as a dispositional psychological characteristic is a strong and positive predictor of negative stress (distress), over and above other selected predictors. The distress during the COVID-19 pandemic is higher for people who are chronic worriers, and it is lower for people who have low levels of worry.

It can be concluded, without any doubt, that general dispositional tendencies, in this case, the tendency to worry, are clearly manifested in crises such as the current one connected with COVID-19, and that such personality tendencies contribute to greater distress in individuals. Particularly surprising is the relatively low degree of importance of social support from friends in overcoming distress.

## 9. Limitations of This Study

Although these findings are important for understanding the interplay between different personal and social factors that could have some protective or risk role in experiencing distress, there are some limitations that should be noted. These correlational data do not allow for conclusions that might be related to cause and effect. Our data could also be analyzed through a hypothetical model in which fear of COVID-19 could be a mediator between the estimation of the spread of COVID-19 and distress.

The survey involved primarily young people and those who are familiar with the use of online platforms and social networks. Additional studies should ensure more representative involvement across the population.

An additional limitation of the study stems from the non-longitudinal design. The research was undertaken during the first wave of the COVID-19 pandemic. The highly uncertain situation of a prolonged pandemic crisis poses additional challenges regarding its consequences. Therefore, follow-up studies in different phases of the ongoing pandemic are needed.

Finally, the impact of other variables was not considered due to the time limitations on an online survey. The selected variables explained about 50–67% variance of distress which indicates that, in future studies, we should consider other risk or protective factors in order to create recommendations for improving preventive programs and policies.

## Figures and Tables

**Table 1:** Model Fit Indices and Model comparisons for MG-CFA.

	*Χ* ^2^	*df*	*p*	CFI	RMSEA
Configural	736.703	201	<0.001	0.920	0.085
Metric	661.429	209	<0.001	0.932	0.077
Scalar	1215.682	225	<0.001	0.852	0.112
	**Δ*χ*^2^**	**Δ*df***	***p***	**ΔCFI**	**ΔRMSEA**
Metric vs. Configural				0.012	−0.008
Scalar vs. Metric	554.253	16	<0.001	−0.080	0.035
Scalar vs. Configural	478.979	24	<0.001	−0.068	0.027

**Table 2:** Summary statistics of variables (Italy *n* = 491; Serbia *n* = 297; Romania *n* = 312).

Italy				Serbia			Romania		
	*M*	*SD*	*α*	*M*	*SD*	*α*	*M*	*SD*	*α*
1. Distress	2.73 a	0.84	0.87	2.61 a	0.90	0.87	2.35 b	0.83	0.88
2. Fear from COVID-19	2.90 a	1.00	0.90	2.46 b	1.01	0.89	3.30 c	1.06	0.89
3. Widespread COVID-19	2.22 a	0.77	-	1.73 b	0.98	-	2.76 c	1.14	-
4. Tendency to worry	2.96 a	0.88	0.90	2.93 a	0.98	0.91	2.52 b	0.99	0.93
5. Social support from family	3.60 a	1.16	-	3.68 a	1.12	-	4.10 b	0.85	-
6. Social support from friends	3.53 a	1.11	-	3.36 b	1.06	-	3.24 b	0.98	-
7. Household climate	1.98 a	0.79	0.69	2.00 a	0.93	0.80	1.91 a	0.65	0.33
8. Economic difficulties	3.13 a	0.82	-	3.05 a	0.72		2.82 b	0.83	-
9. Age	29.44 a	14.07	-	29.29 a	14.37	-	31.47 b	10.71	-
10. Trust in institutions	3.10 a	0.93	0.73	2.57 b	1.02	0.80	2.71 b	0.98	0.77

Legend: *α* = Cronbach’s alpha; *M* = means on a scale from 1–5 (except for age). a, b, and c are letters assigned in post-hoc test—groups with the same letter are not significantly different.

**Table 3:** Correlations between variables (Italy *n* = 491; Serbia *n* = 297; Romania *n* = 312).

	1	2	3	4	5	6	7	8	9	10
1. Distress	-									
2. Fear from COVID-19										
Italy	0.45 **	-								
Serbia	0.37 **	-								
Romania	0.50 **	-								
3. Tendency to worry										
Italy	0.66 **	0.41 **	-							
Serbia	0.61 **	0.33 **	-							
Romania	0.74 **	0.32 **	-							
4. Social support from family										
Italy	0.02	0.22 **	0.08	-						
Serbia	−0.16 **	0.03	0.02	-						
Romania	−0.16 **	−0.02	−0.07	-						
5. Social support from friends										
Italy	0.00	0.14 **	0.02	0.43 **	-					
Serbia	−0.11	0.10	0.01	0.52 **	-					
Romania	−0.07	0.09	−0.02	0.47	-					
6. Negative household climate										
Italy	0.16 **	0.09 *	0.20 **	−0.06	−0.13 **	-				
Serbia	0.31 *	0.12 *	0.24 **	−0.20 **	−0.18 **	-				
Romania	0.12 *	0.03	0.13 *	−0.10	−0.10	-				
7. Estimation of COVID-19 widespread in district										
Italy	0.07	0.20 **	0.07	0.08	0.03	0.03	-			
Serbia	0.10	0.15 **	0.03	0.01	−0.03	0.06	-			
Romania	0.13 *	0.25 **	0.07	0.05	0.11	0.03	-			
8. Economic difficulties										
Italy	0.14 **	0.07	0.06	−0.05	−0.14 **	0.10 *	0.10 *	-		
Serbia	0.28 **	0.04	0.14 *	−0.17 **	−0.27 **	0.16 **	−0.06	-		
Romania	0.16 **	−0.01	0.12 *	−0.07	−0.16 **	0.07	−0.01	-		
9. Age										
Italy	−0.17 **	−0.06	−0.20 **	−0.00	−0.23 **	−0.20	−0.04	−0.01	-	
Serbia	−0.19 **	−0.12	−0.17 **	−0.08	−0.11	−0.09	−0.08	0.17 **	-	
Romania	−0.18 **	−0.02	−0.30 **	−0.22 **	−0.12	−0.21 **	−0.03	0.06	-	
10. Gender										
Italy	0.24 **	0.18 **	0.18 **	−0.03	0.08	−0.02	0.02	0.01	−0.09	-
Serbia	0.06	0.05	0.05	0.11	0.07	−0.16 **	0.02	−0.11	−0.23 **	-
Romania	0.21 **	0.16 **	0.21 **	0.02	0.06	0.08	0.06	0.12 *	−0.09	-
11. Level of education										
Italy	−0.09	−0.00	−0.11	0.01	−0.06	−0.13 **	0.13 **	−0.13	0.38 **	−0.06
Serbia	−0.19 **	−0.06	−0.12 *	0.09	0.04	−0.13 *	0.05	−0.14 *	0.28 **	−0.06
Romania	−0.04	0.09	−0.07	0.04	0.08	−0.07	0.20 **	−0.15 *	0.23 **	−0.03
12. Marital status										
Italy	−0.10 *	−0.01	−0.13 *	0.09	−0.12 *	−0.14 *	−0.01	−0.02	0.61 **	0.21 **
Serbia	−0.15 *	−0.05	−0.17 **	−0.08	−0.12 *	−0.18 **	0.10 *	0.07	0.69 **	0.25 **
Romania	−0.19 **	−0.07	−0.27 **	0.01	−0.01	−0.08	0.01	−0.03	0.41 **	0.14 *
13. Having been distant from family										
Italy	−0.03	−0.03	−0.04	0.05	−0.14 *	−0.14 *	0.07	0.03	0.36 **	−0.03
Serbia	−0.06	−0.01	−0.04	0.12 *	−0.09	−0.04	0.03	0.09	0.19 *	−0.05
Romania	0.09	0.12 *	0.05	0.01	0.05	−0.03	0.10	0.05	0.02	−0.12 *
14. Trust in institutions										
Italy	0.10 *	0.12 *	0.17 **	0.20 **	−0.05	−0.02	−0.14 *	−05	−0.07	0.02
Serbia	−0.04	0.14 *	0.05	0.12 *	0.28	−0.07	0.01	−0.22	0.0.01	−0.05
Romania	0.02	0.29 **	−0.05	0.11	0.21 **	−0.01	0.04	−0.21 **	−0.03	−0.05

Note. Gender: male = 0, female = 1; * *p <* 0.05, ** *p <* 0.01.

**Table 4:** Results of multiple regression analysis.

STEP1	Italy			Serbia			Romania		
	*β*	*t*	*p*	*β*	*t*	*p*	*β*	*t*	*p*
Gender	0.09	2.55	0.01	0.04	0.87	n.s.	0.04	1.18	n.s.
Age	−0.13	−2.69	0.007	−0.07	−1.14	n.s.	−0.01	−0.23	n.s.
Level of education	−0.01	−0.01	n.s.	−0.07	−1.41	n.s.	−0.03	−0.71	n.s.
Marital status	−0.01	−0.04	n.s.	0.01	0.10	n.s.	−0.02	−0.42	n.s.
Economic difficulties	0.10	2.97	0.002	0.15	3.27	0.001	0.06	1.58	n.s.
WidespreadCOVID-19	−0.01	−0.10	n.s.	0.05	1.20	n.s.	0.02	0.44	n.s.
Fear of COVID-19	0.20	5.18	0.001	0.16	3.42	0.001	0.35	7.88	0.001
Social support from family	−0.05	−1.18	n.s.	−0.10	−1.90	0.05	−0.12	−2.81	0.005
Social support from friends	−0.02	−0.52	n.s.	−0.02	−0.39	n.s.	−0.03	−0.74	n.s.
Household climate	0.02	0.54	0.05	0.11	2.25	0.03	−0.01	−0.24	n.s.
Being distant from family	0.02	0.66	n.s.	−0.03	−0.70	n.s.	0.04	0.97	n.s.
Trust in governmental institutions	−0.01	−0.21	n.s.	−0.04	−0.88	n.s.	0.01	0.14	n.s.
Tendency to worry	0.52	13.55	0.001	0.48	10.08	0.001	0.59	14.11	0.001
Widespread COVID-19 × fear	−0.06	−1.58	n.s.	0.04	0.67	n.s.	0.07	1.63	n.s.
Widespread COVID-19 × worry	0.01	0.31	n.s.	0.02	0.42	n.s.	−0.02	−0.40	n.s.
Widespread COVID-19 × Support family	−0.01	−0.04	n.s.	−0.08	−1.47	n.s.	−0.01	−0.21	n.s.
Widespread COVID-19 × support friends	−0.02	−0.32	n.s.	0.05	0.88	n.s.	0.02	0.42	n.s.
Widespread COVID-19 × trust	−0.04	−1.17	n.s.	0.05	0.98	n.s.	−0.03	−0.86	n.s.
Fear of COVID-19 × worry	0.08	2.26	0.02	0.12	2.83	0.005	0.08	2.19	0.03
Fear of COVID-19 × support family	−0.10	−2.72	0.01	0.01	0.21	n.s.	0.08	1.88	n.s.
Fear of COVID-19 × support friends	0.05	1.37	n.s.	−0.02	−0.38	n.s.	−0.06	−1.44	n.s.
Fear of COVID-19 × trust	−0.02	−0.41	n.s.	−0.05	−0.98	n.s.	0.05	1.36	n.s.

Note. Gender: male = 0, female = 1.

## Data Availability

The datasets pertaining to this study are available from the corresponding author upon request.

## Supplementary Materials

The following are available online at https://www.mdpi.com/article/10.3390/ijerph18147261/s1, Table S1. Principal Component Analysis and Component Loadings of the.
