# Supplementary material for: A Cross-Cultural Study of Distress during COVID-19 Pandemic: Some Protective and Risk Factors
Source: Int J Environ Res Public Health. 2021 Jul 7;18(14):7261. doi: 10.3390/ijerph18147261 (PMC8305545; doi:10.3390/ijerph18147261)
Supplement: Supplementary file 1 [file ijerph-18-07261-s001.zip › ijerph-1190172-supplementary.pdf]

Supplements

Table S1. Principal Component Analysis and Component Loadings of the

Items of the COVID-19 fear scale	PC1
I am afraid that I might get the Coronavirus.	.85
I am afraid that I may end up in intensive care because of COVID-19.	.84
I am afraid that I might die if I get the Coronavirus infection.	.79
I am afraid that a loved one might get the Coronavirus infection.	.83
I am afraid that someone in my family might end up in hospital because of COVID-19.	.85
